# Gender disparities in multi-state health transitions and life expectancy among the ≥50-year-old population: A cross-national multi-cohort study

**DOI:** 10.7189/jogh.14.04156

**Published:** 2024-09-06

**Authors:** Zuliyaer Talifu, Shuai Guo, Binbin Su, Yu Wu, Yunhe Wang, Jufen Liu, Yanan Luo, Xiaoying Zheng

**Affiliations:** 1 School of Population Medicine and Public Health, Chinese Academy of Medical Sciences & Peking Union Medical College, Beijing, China; 2 Nuffield Department of Population Health, University of Oxford, Oxford, UK; 3 Department of Epidemiology and Biostatistics, School of Public Health, Peking University, Beijing, China; 4 Department of Global Health, School of Public Health, Peking University, Beijing, China; 5 APEC Health Science Academy, Peking University, Beijing, China

## Abstract

**Background:**

Understanding how disability progresses with ageing is important for shaping policies aimed at improving older adults’ quality of life, especially when considering the global trends in ageing, life expectancy (LE), and gender disparity. We aimed to assess the health transition probabilities of daily living activities and their implications on LE and gender gaps in global middle-aged and elderly populations.

**Methods:**

In this multi-cohort study with a sample of 74 101 individuals aged ≥50 years, we analysed data from six international cohorts: the China Health and Retirement Longitudinal Study (CHARLS), the English Longitudinal Study of Ageing (ELSA), the Health and Retirement Study (HRS) in the USA, the Mexican Longitudinal Study of Ageing (MHAS), the Korean Longitudinal Study of Ageing (KLoSA), and the Survey of Health, Ageing and Retirement in Europe (SHARE). We estimated probabilities between robust health; disabilities related to instrumental activities of daily living (IADL) and basic activities of daily living (BADL); and mortality through multi-state Markov models. We included gender as a covariate in the models to calculate hazard ratios (HRs), while we calculated LE within the distinct health states of robust health, IADL disabilities, BADL disabilities, and mortality using the stochastic population analysis for complex events (SPACE) microsimulation.

**Results:**

Women had higher progressions to disability (IADL: HR = 1.392; BADL: HR = 1.356) compared to men, who conversely showed lesser progression from IADL to BADL disability (HR = 0.856) and lower mortality rates (span of HRs = 0.232–0.692). LE at age 50 favoured women (32.16–38.22 years) over men (28.99–33.58 years), yet they spent more time in states of disability. We otherwise observed significant regional and gender disparities in healthy LE.

**Conclusions:**

We identified ageing patterns in which longer lives are often coupled with extended periods of disability. Pronounced gender and regional differences indicate a need for targeted health interventions to address inequities and improve seniors’ quality of life. Our findings highlight the necessity for policy interventions focussed on health equity to more completely respond to the demographic shift towards older populations.

Although population ageing has been a key accomplishment of societal progress, it also resulted in longer periods of life being spent with illnesses, resulting in a growing proportion of public health expenditure for elderly care [1]. For the general population and policymakers alike, life expectancy (LE) serves as a tangible and comprehensive metric of overall health and longevity [2]. The global average LE has increased from 46.5 years in 1950 to 73.4 years in 2023 (76.0 years for women and 70.8 years for men, with a gender difference of up to 5.2 years) [3]. Despite this increase, the evolving disease landscape and extended periods of living with illness and disability indicate that LE alone may not adequately reflect health quality, with up to 75% of an individual’s later years potentially spent in frailty or pre-frailty [4,5]. With growing emphases on healthy ageing, but especially following endorsement by the World Health Organization (WHO) [6], metrics like healthy LE and disability-free LE have become increasingly relevant in public discourse.

The study of health trajectories in the elderly population is essential for healthcare spending analysis, social planning, labour force trend forecasting, and retirement policy development [1]. The human life course often follows an inverted U-shaped curve, with peak functional capacity in youth gradually declining with age [6], progressing from a healthy state to limited independence and (eventually) significant self-care disability [7]. Recognising and addressing this trajectory of decline is essential in shaping effective public health interventions tailored to the specific needs of ageing populations [8]. In contrast to static correlational studies, techniques such as multi-state Markov models and multi-state life tables allow us to observe dynamic changes across different states of health [9,10]. This enables a more nuanced understanding of the transitions between various stages of health and disability over time, offering insights into the progression of ageing and disease. By exploring disability patterns in the elderly, we can determine key demographic groups and critical intervention windows where targeted strategies can have the most significant impact, thereby guiding resource allocation and informing the development of timely and relevant interventions to promote healthy ageing and improve elderly individuals’ quality of life [1].

Disparities in ageing, disease prevalence, socioeconomics, and healthcare policies contribute significantly to global variations in the survival rates of older adults [11]. For this reason, objective indicators like activities of daily living became commonly used in cross-cultural comparisons when measuring disability-free LE [12]. Notably, individuals with disabilities in instrumental activities of daily living (IADL) and basic activities of daily living (BADL) have distinct intervention requirements, focussing on independent living abilities and essential personal activities, respectively [13]. However, existing research has mainly focussed on overall LE, disability-free LE, or chronic disease-free LE [14–16], overlooking a comprehensive evaluation of the life trajectories of elderly populations worldwide. There are also few comprehensive studies on IADL, BADL, mortality trends, and gender differences overall.

In response to this research gap, we designed a multicohort study to investigate the transitions between IADL disability, BADL disability, and mortality among middle-aged and older populations across diverse regions and genders, thereby addressing existing research voids. We also sought to examine LE patterns for middle-aged and older individuals based on region and gender while considering their statuses of ‘robust’, IADL disability, and BADL disability.

## METHODS

### Study design and participants

We included six longitudinal cohorts from 18 countries (Figure S1 in the **Online Supplementary Document**): the China Health and Retirement Longitudinal Study (CHARLS), waves 1–4; the English Longitudinal Study of Ageing (ELSA), waves 3–6; the Health and Retirement Study (HRS), waves 10–14; the Korean Longitudinal Study of Ageing (KLoSA), waves 3–7; the Mexican Health and Aging Study (MHAS), waves 3–5, and the Survey of Health, Ageing and Retirement in Europe (SHARE), waves 4–8. All these cohorts are part of The Health and Retirement Study family, a collective of international studies inspired by the original HRS – a longitudinal and multidisciplinary survey of adults over age 50 initiated in 1992 and conducted biennially with refreshers to remain nationally representative.

We selected these cohorts because we required high-quality longitudinal data on middle-aged and elderly populations. The six cohorts are known for their rigorous data collection methodologies and multiple follow-up waves, which makes their data reliable and comparable, and they are representative of several national populations and diverse socioeconomic and cultural contexts. Importantly, they each have at least three survey waves, which was a prerequisite of our multi-state model approach. Relatedly, we selected waves 3–6 (years 2007–2013) of the ELSA study due to the availability of mortality data linked up to March 2013 [17] to ensure methodological consistency and data comparability with the other cohorts, aligning with the standards of longitudinal analysis and improving the validity of our findings [18].

The participants in all cohorts were informed about their objectives, procedures, potential risks, and benefits. They had to provide consent before participation, which ensured that they understood their rights, including the voluntary nature of participation and the confidentiality of their data. Each regional ethics committe reviewed and approved these consent procedures for each cohort to ensure they met international ethical standards.

The response rates for each cohort wave ranged from 62.6% to 89.1% (Table S1 in the **Online Supplementary Document**). We focussed on individuals aged ≥50 years, as this age group commonly experiences chronic health conditions and medical issues, impacting both their LE and the quality of those years lived, which are core components of the concept of healthy LE.

### Procedures and outcomes

The researchers in the original cohorts surveyed participants in each group about their difficulties with daily activities related to disabilities, such as dressing; bathing; eating; getting out of bed; squatting and standing; and controlling urination and defecation. IADLs are activities needed for independent living, like housework, cooking, shopping, using the phone, managing medication, handling finances, and reading maps.

We considered individuals to have an IADL or BADL disability if they could not do or struggled with one or more IADL or BADL, respectively. Based on these criteria, we classified them into four states: robust, with no BADL or IADL disability; IADL disability, indicating at least one limitation in IADLs, but no limitation in BADLs; BADL limitation, indicating at least one limitation in BADLs, including cases where IADL limitation is also present; and death (Figure S2 in the **Online Supplementary Document**).

We assessed mortality by recording the time of death in follow-up interviews and using linked register data. For participants with missing death records, especially for the CHARLS waves 3–5, we assumed that deaths occurred evenly between the two survey waves. Taking the CHARLS waves as an example, we used the midpoint between the survey dates of wave 3 and wave 5 as the estimated time of death [19,20]. We also conducted sensitivity analyses to address the potential uncertainty and variability in the estimated dates of death by adjusting the estimated year of death to earlier or later dates within the interval to evaluate how they might affect the study outcomes. The results indicated that the impact on LE was minimal, with variations of less than 0.1 years across different adjustment methods (data not shown).

### Multi-state Markov model assumptions

Our primary hypothesis is that the health trajectories of middle-aged and elderly individuals follow a Markov process, where transitions between different health states are determined primarily by the individual’s current state of health and are independent of the history of previous states. In applying the multi-state Markov model to our research, we also hypothesised that gender significantly influences the transitions between different health states, whereby we expected to observe gender-specific differences in the probabilities and patterns of transitions, reflecting distinct health trajectories for men and women as they age. More precisely, by incorporating gender as a covariate in the model, we wanted to investigate how gender impacts the intensity and dynamics of health state transitions, providing insights into gender-specific disparities in health outcomes among middle-aged and elderly populations.

### Statistical analysis

We employed a multi-state Markov (MSM) model in R. version 4.3.0 (R Core Team, Vienna, Austria). The MSM is a commonly used method for describing the progression of health states over time [21]. It therefore allowed us to estimate the expected duration of each state, the probability of transitions between them, and the risk ratio of transitions across groups. Using proportional hazard models, we performed parameter estimation via maximum likelihood estimation to derive the transition probabilities of health states for different subgroups. We included gender as a covariate to estimate the hazard ratios (HRs) for gender on transition intensity, which measures the relative risk of an event occurring at any given point in one group compared to another. We further calculated confidence intervals (CIs) assuming a normal distribution for the log effect.

We used the Stochastic Population Analysis for Complex Events (SPACE) programme in SAS, version 9.4 (SAS Institute, Cary, NC, USA) to calculate lifespans in different states [10]. We adjusted for age and gender using multinomial logistic regression models. To simulate health transitions from age 50 to 90 years, we employed a random microsimulation technique to generate a cohort of 100 000 individuals, with the intent of estimating the average remaining lifespan and the years with or without IADL/BADL disability within specific age brackets.

## RESULTS

### Baseline characteristic

We included 74 101 participants in our study (CHARLS: n = 10 394; ELSA: n = 7420; HRS: n = 14 875; KLoSA: n = 7246; MHAS: n = 6660; SHARE: n = 27 506) (Figure S3 and Table S2 in the **Online Supplementary Document**). There were more women than men in all cohorts, while the mean age of participants at baseline varied from 62.42 to 69.53 (**Table 1**). These variations in age meant that we had to consider age-related factors when interpreting transition probabilities, while the higher proportion of women across cohorts suggests potential gender-specific health trajectories, with women potentially facing higher risks of certain disabilities.

**Table 1:** Baseline characteristics of study participants*

	Number of individuals included at baseline			Age in years at baseline						Disability status at baseline			
**Cohort**	**Total**	**Men**	**Women**	**Age, x̄ (SD)**	**50–59**	**60–69**	**70–79**	**80–89**	**≥90**	**No limitation**	**IADL disability**	**BADL disability**	**Deaths during follow-up**
CHARLS	10 394	5174 (49.78)	5220 (50.22)	62.42 (8.44)	4525 (43.53)	3762 (36.19)	1667 (16.04)	405 (3.90)	35 (0.34)	7119 (69.26)	1326 (12.76)	1869 (17.98)	1337 (12.86)
ELSA	7420	3343 (45.05)	4077 (54.95)	65.75 (10.74)	2587 (34.87)	2250 (30.32)	1677 (22.60)	785 (10.58)	121 (1.63)	5466 (73.67)	537 (7.19)	1417 (19.10)	881 (11.87)
HRS	14 875	6355 (42.72)	8520 (57.28)	66.95 (11.17)	4846 (32.58)	3810 (25.61)	4001 (26.90)	1823 (12.26)	395 (2.66)	10 195 (68.54)	1748 (11.75)	2932 (19.71)	3653 (24.56)
KLoSA	7246	3151 (43.49)	4095 (56.51)	65.89 (10.18)	2289 (31.59)	2267 (31.29)	1924 (26.55)	678 (9.36)	88 (1.21)	6297 (86.90)	620 (8.56)	329 (4.54)	1526 (21.06)
MHAS	6660	2781 (41.76)	3879 (3879)	69.53 (8.11)	515 (7.73)	3151 (47.31)	2167 (32.54)	733 (11.01)	94 (1.41)	5175 (77.70)	375 (5.63)	1110 (16.67)	1320 (19.82)
SHARE	27 506	11 954 (43.46)	15 552 (56.54)	66.17 (9.88)	8026 (29.18)	9583 (34.84)	6909 (25.12)	2720 (9.89)	268 (0.97)	21480 (78.09)	258 (9.38)	3445 (12.52)	3928 (14.28)

BADL – basic activities of daily living, CHARLS – China Health and Retirement Longitudinal Study, ELSA – English Longitudinal Study of Ageing, HRS – Health and Retirement Study, IADL – instrumental activities of daily living, KLoSA – Korean Longitudinal Study of Ageing, MHAS – Mexican Longitudinal Study of Ageing, SD – standard deviation, SHARE – Survey of Health, Ageing and Retirement in Europe, x̄ – mean

*Presented as n (%) unless specified otherwise.

At baseline, a significant proportion of individuals (ranging from 68.54% to 86.90% among the cohorts) had no limitations in IADL or BADL, which is why we categorised them as ‘robust’. Conversely, 5.63% to 12.76% experienced IADL disability, while 4.54% to 19.71% experienced BADL disability. These baseline characteristics served us as essential indicators of initial health status, with implications for the trajectory of health transitions and the development of targeted interventions. The mortality rate during the follow-up period between the cohorts ranged from 11.87% to 24.56%, suggesting that baseline health, healthcare quality, and socioeconomic conditions may significantly influence long-term outcomes.

### Longitudinal state transitions

**Figure 1:**
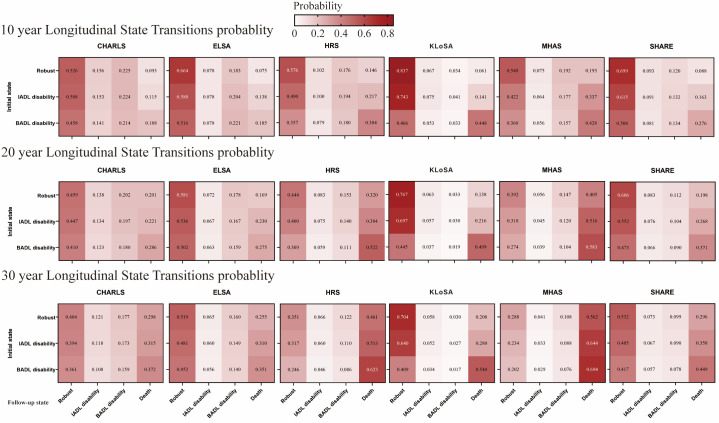
Heat maps based on the 10/20/30-year state transition probabilities for each cohort. The horizontal axis corresponds to the initial states (‘robust’, ‘IADL-disabled’, and ‘BADL-disabled), while the vertical axis represents the final states (three initial states or death). Each row represents a 10-year, 20-year, 30-year time span. Darker shades in the table indicate higher probabilities of transitioning to the respective state, with each cell containing a specific probability of transitioning to that state.

To ensure the comparability of state transition probabilities, we assessed the probabilities of transitioning from the baseline health states of ‘robust’, ‘IADL-disabled’, and ‘BADL-disabled’ to these same states and death over 10, 20, and 30-year periods (**Figure 1**).

The likelihood of developing IADL disabilities after 10 years was higher for the CHARLS cohort (14.1% to 15.6%) compared to the non-CHARLS cohorts (5.3% to 10.2%). The probability of transitioning to BADL disability within ten years was consistent across multiple cohorts, ranging from 15.7% to 22.9% for most cohorts and being slightly lower at 12.0% to 13.4% for the SHARE cohort. The KLoSA cohort had the lowest chance of developing BADL disability over 10 years, with a probability ranging from 3.3% to 4.1%. The likelihood of experiencing IADL and BADL disabilities also decreased slightly over 20 and 30 years.

Transition probabilities to mortality varied significantly based on initial health conditions. Over 10 years, individuals in optimal health had mortality risks ranging from 7.0% to 19.3%, while those with BADL disabilities faced much higher risks, ranging from 18.8% to 44.8%. This doubling of mortality risk highlights the increased vulnerability of individuals with disabilities. After 20 years, mortality rates were 13.8% to 40.5% for healthy individuals, 21.6% to 51.6% for those with IADL disabilities, and 27.8% to 58.3% for those with BADL disabilities. After 30 years, mortality rates increased again, especially for the MHAS cohort. The KLOSA cohort had a 54.0% mortality rate for those with initial BADL disability – higher than the other cohorts with less than 50% mortality after 30 years.

### Gender disparities

**Figure 2:**
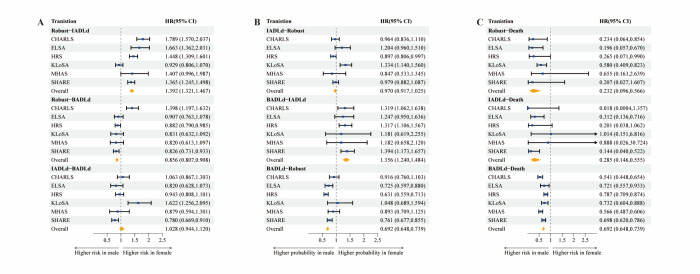
The state transition HRs for different genders (men as reference).

The analysis of HRs for transitioning between different states in relation to gender highlighted distinct patterns. Women had a higher likelihood of transitioning from a ‘robust’ state to being ‘IADL-disabled’, with an HR of 1.392 (95% CI = 1.321–1.467). However, women had a reduced risk of transitioning from a ‘robust’ state to being ‘BADL-disabled’, with an HR of 0.856 (95% CI = 0.807–0.908). We saw no significant gender differences in the progression from being ‘IADL-disabled’ to ‘BADL-disabled’ (HR = 10.28; 95% CI = 0.944–1.120) (**Figure 2**, panel A).

We also saw gender differences in the recovery from disability. Women had a higher likelihood of transitioning from being ‘BADL-disabled’ to being ‘IADL-disabled’, with an HR of 1.356 (95% CI = 1.240–1.484). However, they had a lower likelihood than men of transitioning from being ‘BADL-disabled’ to a ‘robust’ state, with an HR of 0.692 (95% CI = 0.648–0.739). We observed no significant gender difference in transitioning from being ‘IADL-disabled’ to a ‘robust’ state (HR = 0.970; 95% CI = 0.917–1.025) **(****Figure 2****,** panel B).

The pattern of transition to death varied based on gender. Women consistently exhibited a lower risk of mortality compared to men across multiple states: from robust to death (HR = 0.232; 95% CI = 0.096–0.566), from IADL disability to death (HR = 0.285; 95% CI = 0.146–0.555), and from BADL disability to death (HR = 0.692; 95% CI = 0.648–0.739) (**Figure 2****,** panel C).

### LE analysis

**Figure 3:**
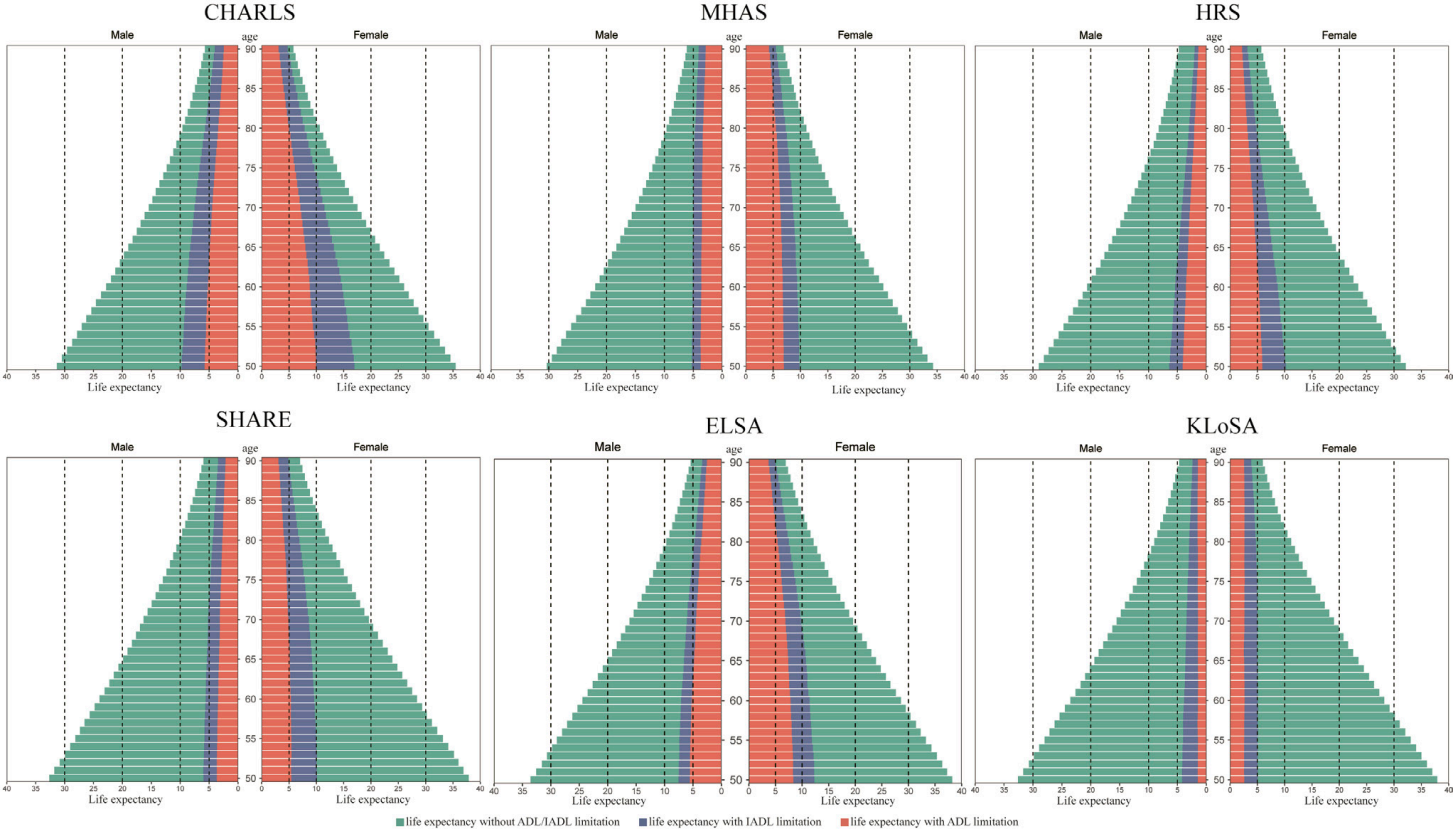
Expected lifespan under different states from 50 to 90.

**Figure 4:**
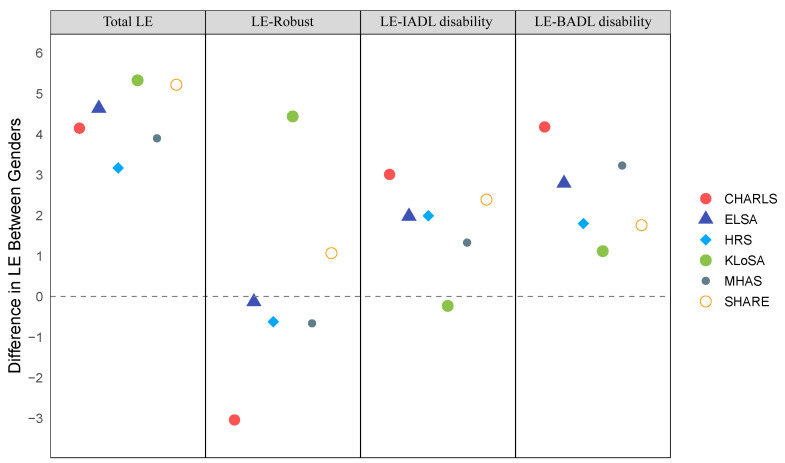
Difference in LE between genders at 50 (men as reference).

We found women generally have longer LE than men, with the smallest gap in the HRS cohort (3.17 years) and the largest in the KLoSA cohort (5.33 years) (**Figure 3**). For example, the LE for 50-year-old women is 35.49 years in China, 38.22 years in the UK, 32.16 years in the USA, 37.92 years in South Korea, 34.24 years in Mexico, and 37.87 years in the EU. For 50-year-old men, the LE is 31.34 years in China, 33.58 years in the UK, 28.99 years in the USA, 32.58 years in South Korea, 30.34 years in Mexico, and 32.65 years in the EU. We also found that women live longer with disabilities, but have a lower overall LE compared to men in the UK, USA, Mexico, and China (**Figure 4**). In general, they tend to have longer periods of life with disability in all cohorts except for South Korea.

Our analysis of time spent in a ‘robust’ state or as ‘IADL-disabled’ and ‘BADL-disabled’ relative to overall LE showed distinct survival patterns among different demographic groups (Figure S4 in the **Online Supplementary Document**). In China, women at age 50 are likely to spend almost half of their remaining LE dealing with disability, with a significant portion attributed to both BADL and IADL disabilities. Women at age 50 have a projected LE of 4.15 years longer than men, but they are estimated to spend 7.2 more years in a disabled state. This trend is consistent across different levels of disability. While Mexican and EU men and women have similar LE distributions at age 50, older Mexican women experience a faster decline in robust LE compared to EU females, with half of their LE spent with disabilities by age 70. The proportion of LE compromised by disability is similar for women in the UK and the USA, but the incidence of BADL disability is higher in the UK. We calculated LE for individuals at ages 50, 60, 70, and 80 in each state of disability, showing that those in IADL and BADL states are more likely to remain in these conditions as they get older (Figure S5–8 in the **Online Supplementary Document**).

## DISCUSSION

As human LE is continually increasing, we aimed to add new evidence to the discussions on ageing and longevity. With this in mind, we analysed LE in relation to different health states, namely robustness, IADL disabilities, and BADL disabilities. We also compared ageing patterns in diverse international contexts by combining data from six cohorts in 18 countries. Our findings suggest that, while disparities in LE at age 50 may not vary much across regions, differences in years lived with disabilities can range up to a decade. Gender-based health inequalities were also evident, with women having longer LE but shorter LE with robustness. These improved LE estimates can help shape policies on ageing, healthcare, and public health interventions by focussing on quality of life in later years, offering insights for scholars and policymakers in the fields of gerontology and public health [22]. Our results suggest the need for more health interventions to prevent or delay disabilities in daily activities and improve management strategies for those already affected

### Key findings

Our study examines how ageing impacts disability transitions, showing differences based on gender and region that affect mortality. Three patterns of ageing have previously been identified: compression of morbidity [2], expansion of chronic diseases and disabilities [23], and a dynamic equilibrium combining elements of both [24]. These patterns can lead to either an absolute or relative increase in LE characterised by disability, meaning an increase in either the number of years lived with a disability or the proportion of life spent with disability. Currently, the gap between men's and women's LE is gradually widening in most countries [25]. In our study of China, the UK, the USA, and Mexico, the difference in LE was primarily driven by a rise in years lived with IADL and BADL disabilities, indicating a trend of 'disability expansion'. Conversely, in the EU and South Korea, the trend aligns more closely with a 'dynamic equilibrium', showing both an increase in robust years and disabled years.

We also conducted the first comprehensive analysis of health trajectories in middle-aged and elderly populations, tracing the progression from a state of robust health to increasing disability in IADL, BADL, and eventually death. Importantly, disability is not static, but rather evolves dynamically over time [6]. By using multi-state models and Markov models as opposed to traditional single-state survival analyses, we gained a more sophisticated understanding of transitions across an individual's lifespan [21]. Our findings indicate that individuals with IADL and BADL disabilities have opportunities for improvement and potential recovery, irrespective of their demographic characteristics. Notably, younger cohorts demonstrate a higher likelihood of positive shifts, leading to decreased disability and increased healthy LE, highlighting the potential benefits of comprehensive interventions. Implementing such initiatives could significantly reduce healthcare costs while promoting long-term quality of life. Consistent with previous research [26,27], we found significant gender disparities contributing to health inequalities, with women being particularly vulnerable to IADL disabilities. Not only do women experience a higher likelihood of IADL disabilities, but they also devote a larger portion of their extended lifespan to managing these impairments compared to men. This gender trend is particularly prominent in middle-income countries, as seen in studies conducted in China and Mexico – regions that have undergone a shift in disease spectrum and healthcare patterns, resulting in a higher likelihood of disabilities occurring, especially in the case of chronic diseases and cancer [28,29]. Additionally, individuals from lower socioeconomic backgrounds face an increased risk of early physical decline, chronic illnesses, and subsequent development of IADL and BADL disabilities [30]. These health disparities highlight the growing need for long-term care services among these populations [31,32].

To mitigate potential biases [33], we evaluated the respondents' abilities in daily life as reported for the three months preceding the survey date in the database. Our findings are consistent with previous research, showing a lower incidence of IADL disability compared to BADL disability [34]. This may be due to the essential nature of BADL tasks and cultural differences in assessing IADL. Preventive measures can delay functional decline and improve quality of life in individuals with IADL disability [35]. Addressing IADL disability through preventive measures could effectively postpone functional deterioration and improve LE. Notably, the KLoSA cohort had a significantly lower baseline incidence of BADL disability. This may be due to how the questionnaire in the KLoSA was designed, leading to potential underreporting of less severe limitations in BADL. This could mean that the cases identified in the KLoSA cohort represent more severe impairment, which is linked to a higher risk of transitioning from disability to death.

### Implications for policy and practice

Our findings support the WHO's emphasis on achievable healthy ageing, highlighting the importance of lifestyle factors in preventing chronic diseases in older adults [36–38]. Creating supportive environments can improve the quality of life for individuals with disabilities, as well as dignity in later stages of life [39]. In this sense, educational and socioeconomic factors play a key role in health behaviours, necessitating targeted interventions to address disparities [40]. The growing elderly population in low- and middle-income countries underscores the need for strong health care and social support systems [41]. Our research emphasises the need for equal access to health resources in regions with higher rates of chronic illnesses, comorbidities, and disabilities. Rehabilitation medicine is seen as a valuable and cost-effective healthcare approach, with an estimated 241 million people worldwide potentially benefiting from these services [42]. Identifying key factors contributing to disability, promoting health behaviour interventions, and improving rehabilitation services can enhance LE and quality of life.

### Limitations

This study has some limitations. First, the categorisation of disability into IADL and BADL may not fully capture the range of limitations in daily living activities, leading to an underestimation or overestimation of the impact of certain disabilities on transition probabilities, potentially skewing the observed patterns [43,44]. Second, although we used data from six globally representative cohort studies, variations exist within each country and region due to the populations’ economic and social statuses, geographic location, time of observation, and access to health care, possibly leading to biased estimates when generalising the results to the entire population of each country, therefore limiting the external validity of our findings [26]. Third, our findings may not reflect global disparities in LE across different countries and regions, particularly in middle and low-income countries with limited availability of longitudinal data, leading to an underrepresentation of populations that might experience different transition dynamics due to distinct socioeconomic and healthcare challenges. Despite these limitations, our use of large cohort data to calculate cross-culturally comparable health indicators for middle-aged and older adults in selected geographic areas provides a basis for health policy assessment and intervention, while also enhancing the robustness of our findings and allowing for a better understanding of health trajectories across diverse contexts.

## CONCLUSIONS

We used multi-state Markov models and SPACE microsimulations to analyse ageing trajectories and identify gender-specific differences and regional variations in disability transitions and mortality among older individuals. We found that women live longer periods of disability, while men progress quickly to high-dependency disability and have higher mortality rates in old age. Balancing longer life expectancies with maintaining a good quality of life is a challenge. It emphasises the importance of adapting healthcare systems and policies to address and manage disabilities and multimorbidity, as well as implementing targeted initiatives to reduce health inequalities. Promoting global inclusivity in ageing therefore involves closing the gap between health span and lifespan to make healthy ageing achievable worldwide. This requires healthcare innovation, societal changes, and international cooperation to address the challenges of an ageing population.

## Additional material


Online Supplementary Document
