# Supplementary material for: Gender disparities in multi-state health transitions and life expectancy among the ≥50-year-old population: A cross-national multi-cohort study
Source: J Glob Health. 2024 Sep 6;14:04156. doi: 10.7189/jogh.14.04156 (PMC11377966; doi:10.7189/jogh.14.04156)
Supplement: Online Supplementary Document [file jogh-14-04156-s001.pdf]

Gender Disparities in multi-state Health Transitions and Life Expectancy among 50+yrs Old Population: A cross-national multi-cohort Study

Online Supplementary Documents

Table S1 The response rates of each wave included in the study cohorts.

Table S2 Baseline data on participants from each country included in the SHARE cohort and mortality during follow-up.

Figure S1 Waves and years of cohorts included in the study.

Figure S2 Flowchart for inclusion and exclusion of study individuals.

Figure S3 Expected lifespan at 50, 60, 70, and 80.

Figure S. Expected lifespan for individuals without ADL limitation at 50, 60, 70, and 80.

Figure S5 Expected lifespan for individuals with IADL disability at 50, 60, 70, and 80.

Figure S6 Expected lifespan for individuals with BADL disability at 50, 60, 70, and 80.

Table S1. The response rates of each wave included in the study cohorts.

	**wave (YEAR)**	**response rate (%)**		**wave (YEAR)**	**response rate (%)**
**CHARLS**	w1(2011)	80.5	**KLoSA**	w3(2010)	81.7
	w2(2013)	88.3		w4(2012)	80.1
	w3(2015)	87.2		w5(2014)	79.2
	w4(2018)	86.5		w6(2016)	78.0
**ELSA**	w3(2007)	73.0		w7(2018)	77.6
	w4(2009)	74.0	**MHS**	w3(2012)	88.1
	w5(2011)	80.0		w4(2015)	88.3
	w6(2013)	78.0		w5(2018)	84.7
**HRS**	w10(2010)	81.0	**SHARE***	w4(2010)	80.5
	w11(2012)	89.1		w5(2012)	82.2
	w12(2014)	87.1		w6(2014)	80.5
	w13(2016)	73.9		w7(2016)	85.3
	w14(2018)	74.4		w8(2018)	62.6

CHARLS-China Health and Retirement Longitudinal Study, ELSA-English Longitudinal Study of Ageing, HRS-Health and Retirement Study, MHAS-Mexican Longitudinal Study of Ageing, KLoSA -Korean Longitudinal Study of Ageing, SHARE-Survey of Health, Ageing and Retirement in Europe, IADL-Instrumental Activities of Daily Living, BADL-Basic Activities of Daily Living

*The response rate of the SHARE is determined by calculating the average retention rates across different waves for each country.

Table S2. Baseline data on participants from each country included in the SHARE cohort and mortality during follow-up.

Country	Number of individuals included at baseline			Age at baseline						Disability status at baseline			Deaths during follow-up n(%)
	N (%)	Male (n, %)	Female (n, %)	Mean age (SD)	50-59 n(%)	60-69 n(%)	70-79 n(%)	80-89 n(%)	≥90 n(%)	No limitation n(%)	IADL Disability n(%)	BADL Disability n(%)	
Total	27,506 (100)	11,954 (43.46)	15,552 (56.54)	66.17 (9.88)	8,026 (29.18)	9,583 (34.84)	6,909 (25.12)	2,720 (9.89)	268 (0.97)	21,480 (78.09)	2,58 (9.38)	3,445 (12.52)	3,928 (14.28)
Austria	2,499 (9.09)	1,041 (41.66)	1,458 (58.34)	65.58 (9.54)	747 (29.89)	906 (36.25)	618 (24.73)	207 (8.28)	21 (0.84)	1,996 (79.87)	253 (10.12)	250 (10.00)	285 (11.40)
Germany	879 (3.26)	428 (47.71)	469 (52.29)	65.89 (7.87)	223 (24.86)	388 (43.26)	238 (26.53)	46 (5.13)	2 (0.22)	737 (82.16)	58 (6.47)	102 (11.37)	68 (7.58)
Sweden	1,195 (3.43)	542 (45.36)	653 (54.64)	69.01 (8.81)	165 (13.81)	526 (44.02)	338 (28.28)	145 (12.13)	21 (1.76)	992 (83.01)	68 (5.69)	135 (11.30)	150 (12.55)
Nether lands	53 (0.19)	34 (64.15)	19 (35.85)	74.02 (9.78)	3 (5.66)	17 (32.08)	17 (32.08)	12 (22.64)	4 (7.55)	32 (60.38)	8 (15.09)	13 (24.53)	53 （100）
Spain	2,504 (9.10)	1,141 (45.57)	1,363 (54.43)	68.81 (10.80)	592 (23.64)	727 (29.03)	724 (28.91)	407 (16.25)	54 (2.16)	1,822 (72.76)	244 (17.49)	438 (17.49)	596 (22.72)
Italy	2,152 (7.82)	979 (45.49)	1,173 (54.51)	66.71 (24.16)	520 (24.16)	798 (37.08)	619 (28.76)	197 (9.15)	18 (0.84)	1,718 (79.83)	185 (8.60)	249 (11.57)	299 (13.86)
France	2,480 (9.02)	1,065 (42.94)	1,415 (57.06)	65.54 (10.30)	841 (33.91)	788 (31.77)	563 (22.70)	265 (10.69)	23 (0.93)	2,006 (80.89)	190 (7.66)	284 (11.45)	266 (10.73)
Denmark	1,601 (5.82)	730 (45.60)	871 (54.60)	64.59 (10.36)	591 (36.91)	549 (34.29)	296 (34.29)	140 (18.49)	25 (1.56)	1,360 (84.95)	122 (7.62)	119 (7.43)	207 (12.93)
Switzerland	2,177 (7.91)	1,014 (46.58)	1,163 (53.42)	64.92 (9.76)	742 (34.08)	764 (35.09)	481 (22.09)	168 (7.72)	22 (1.01)	1,939 (89.07)	109 (5.01)	129 (5.93)	168 （7.72）
Belgium	2,938 (10.68)	1,316 (44.79)	1,622 (55.21)	65.05 (10.34)	1050 (35.74)	944 (32.13)	614 (20.90)	302 (10.28)	28 (0.95)	2,210 (75.22)	286 (9.73)	442 (15.04)	339 (11.54)
Czech Republic	3,109 (11.30)	1,291 (41.52)	1,818 (58.48)	65.85 (8.94)	833 (26.79)	1,301 (41.85)	729 (23.45)	232 (7.46)	14 (0.45)	2,392 (76.94)	394 (12.67)	323 (10.39)	514 (16.53)
Slovenia	1,502 (5.46)	633 (42.14)	869 (57.86)	65.23 (9.92)	521 (34.69)	479 (31.89)	355 (23.64)	139 (9.25)	8 (0.53)	1,171 (77.96)	157 (10.45)	174 (11.58)	183 (12.18)

Estonia	4,399<br>(15.99)	1,740<br>(39.55)	2,659<br>(60.45)	66.78<br>(9.83)	1,198<br>(27.23)	1,396<br>(31.73)	1,317<br>(29.94)	460<br>(10.46)	28<br>(0.64)	3,105<br>(70.58)	507<br>(11.53)	787<br>(17.89)	827<br>(18.80)

SHARE-Survey of Health, Ageing and Retirement in Europe, IADL-Instrumental Activities of Daily Living, BADL-Basic Activities of Daily Living

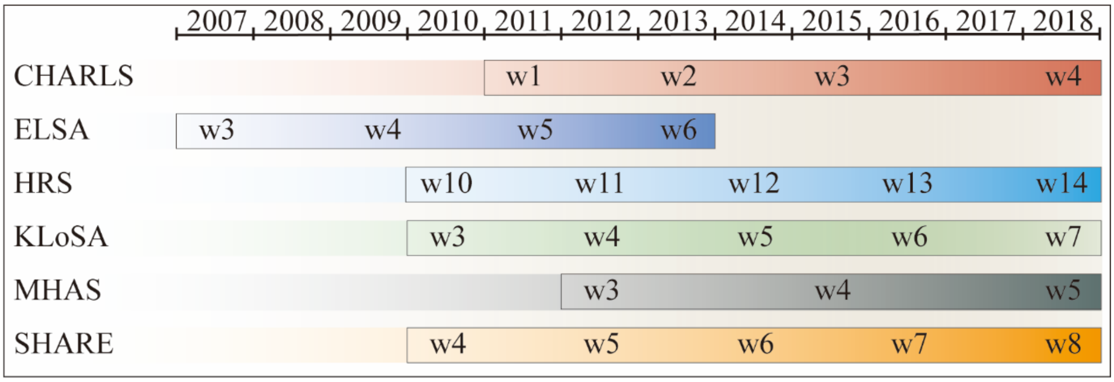

Figure S1. Waves and years of cohorts included in the study.
Abbreviations: China Health and Retirement Longitudinal Study (CHARLS), English Longitudinal Study of Ageing (ELSA), Health and Retirement Study (HRS), Mexican Longitudinal Study of Ageing (MHAS), Korean Longitudinal Study of Ageing (KLoSA), Survey of Health, Ageing and Retirement in Europe (SHARE).

Figure S2. Flowchart for inclusion and exclusion of study individuals.

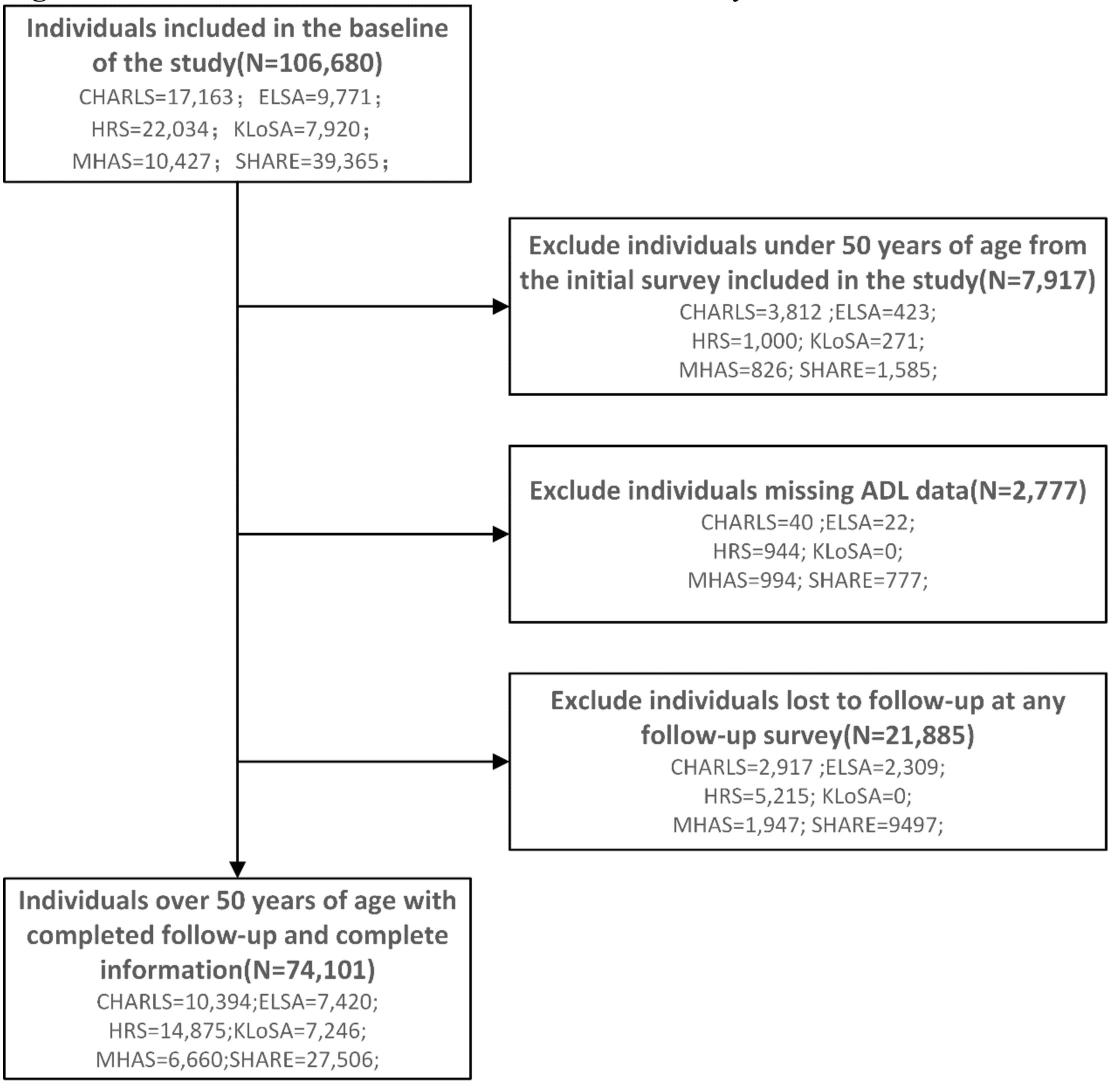

Abbreviations: China Health and Retirement Longitudinal Study (CHARLS), English Longitudinal Study of Aging (ELSA), Health and Retirement Study (HRS), Mexican Longitudinal Study of Aging (MHAS), Korean Longitudinal Study of Aging (KLoSA), Survey of Health, Aging and Retirement in Europe (SHARE).

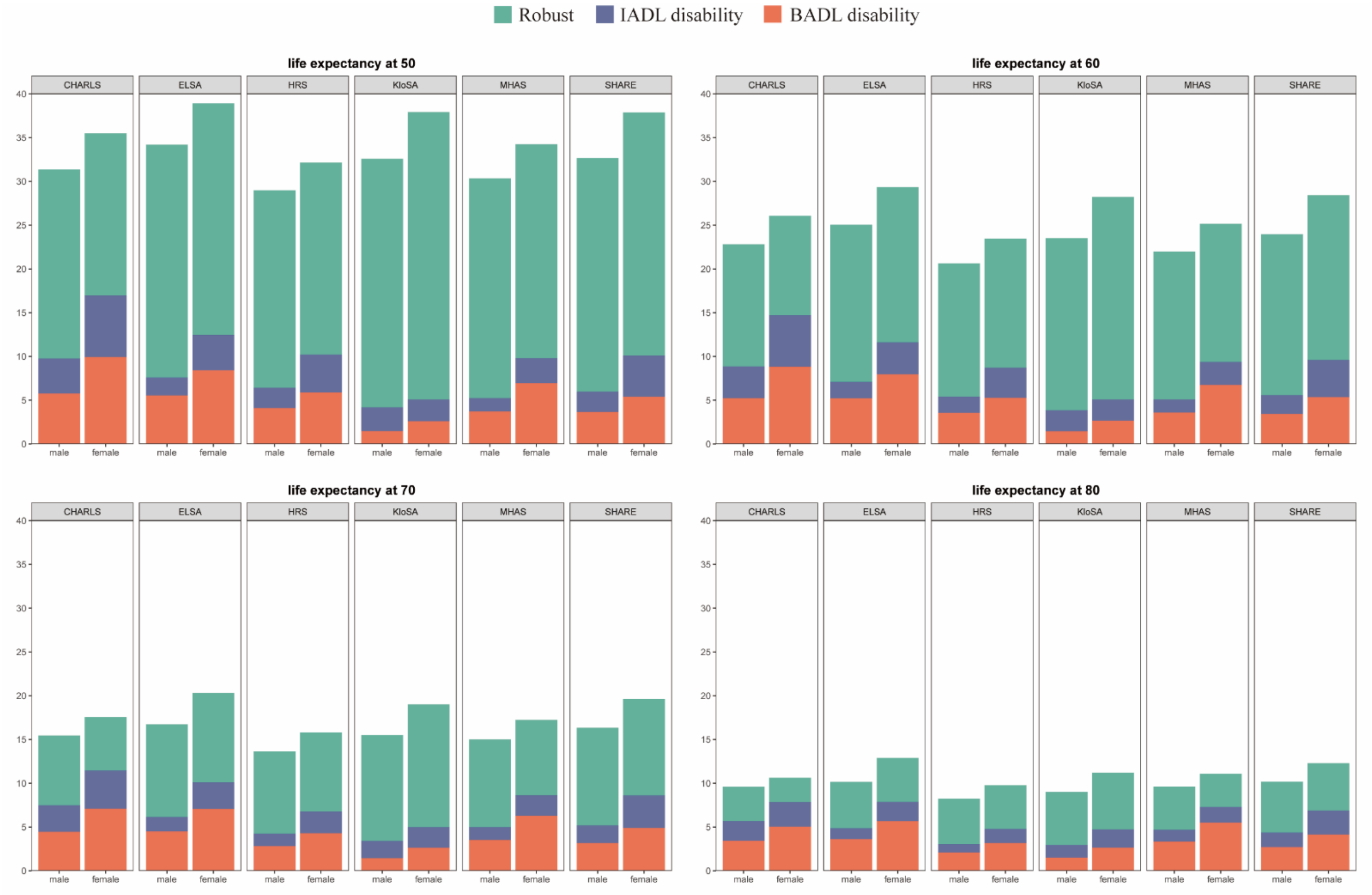

Figure S3. Expected lifespan at 50, 60, 70, and 80.

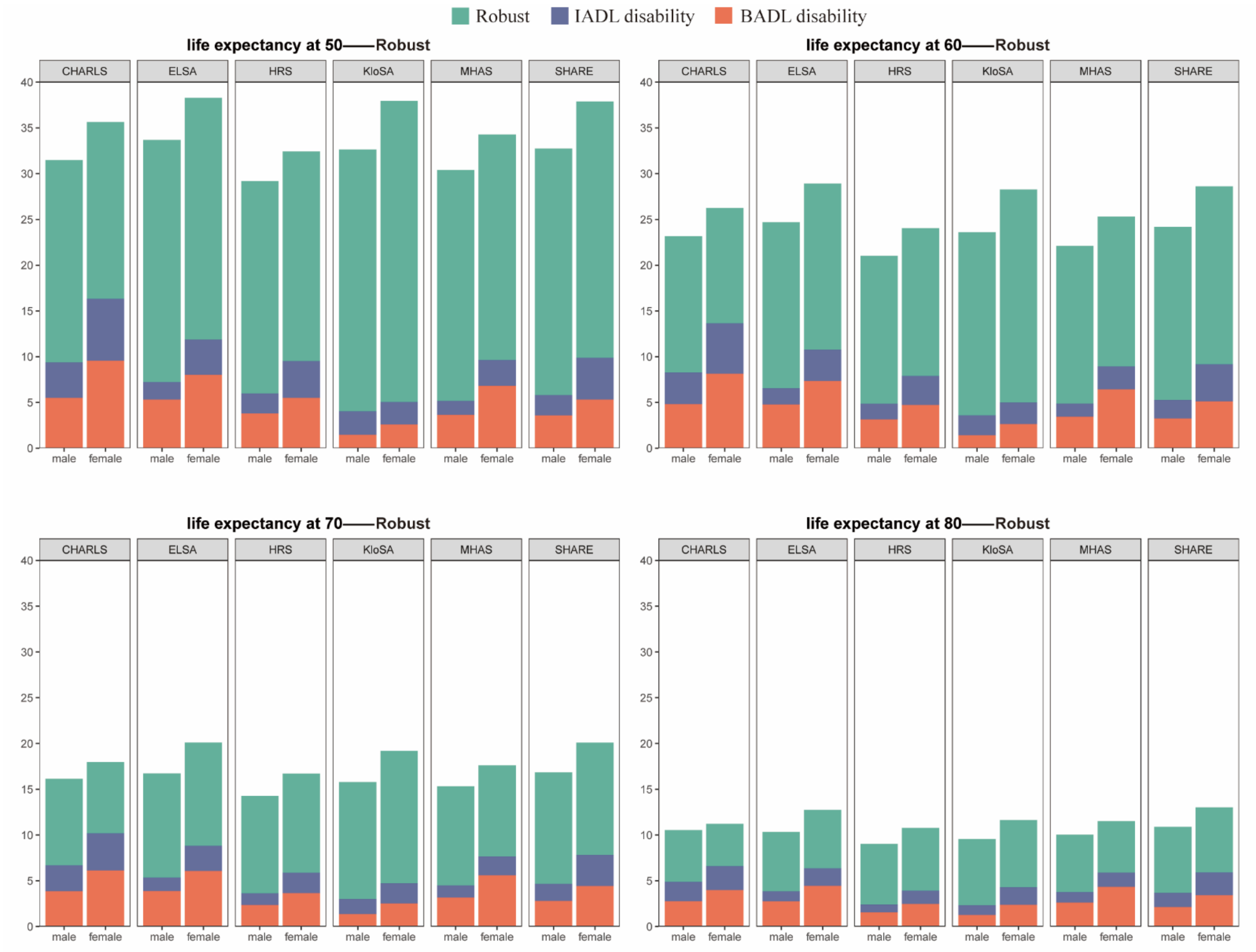

Figure S4. Expected lifespan for individuals without ADL limitation at 50, 60, 70, and 80.

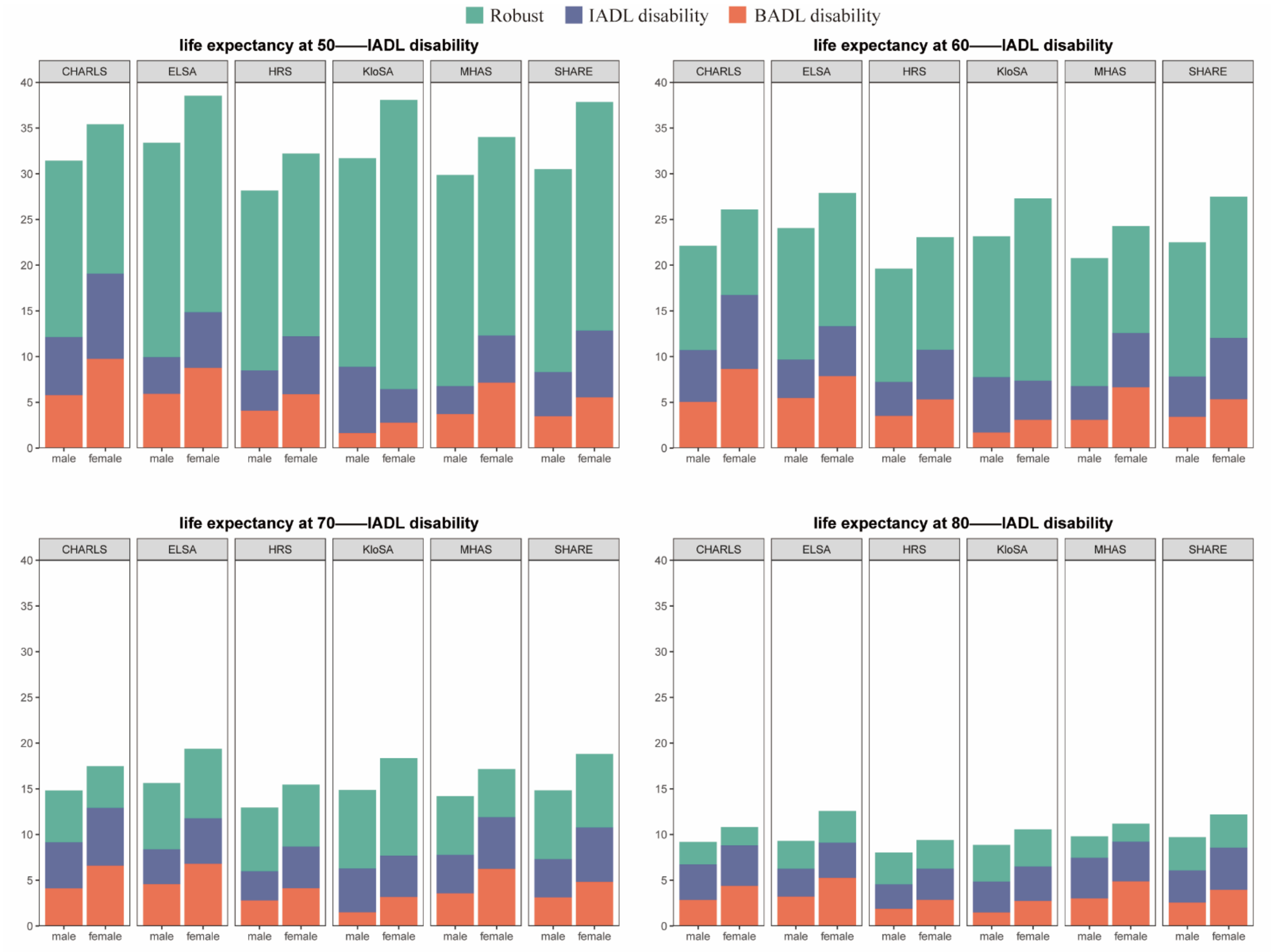

Figure S5. Expected lifespan for individuals with IADL disability at 50, 60, 70, and 80.

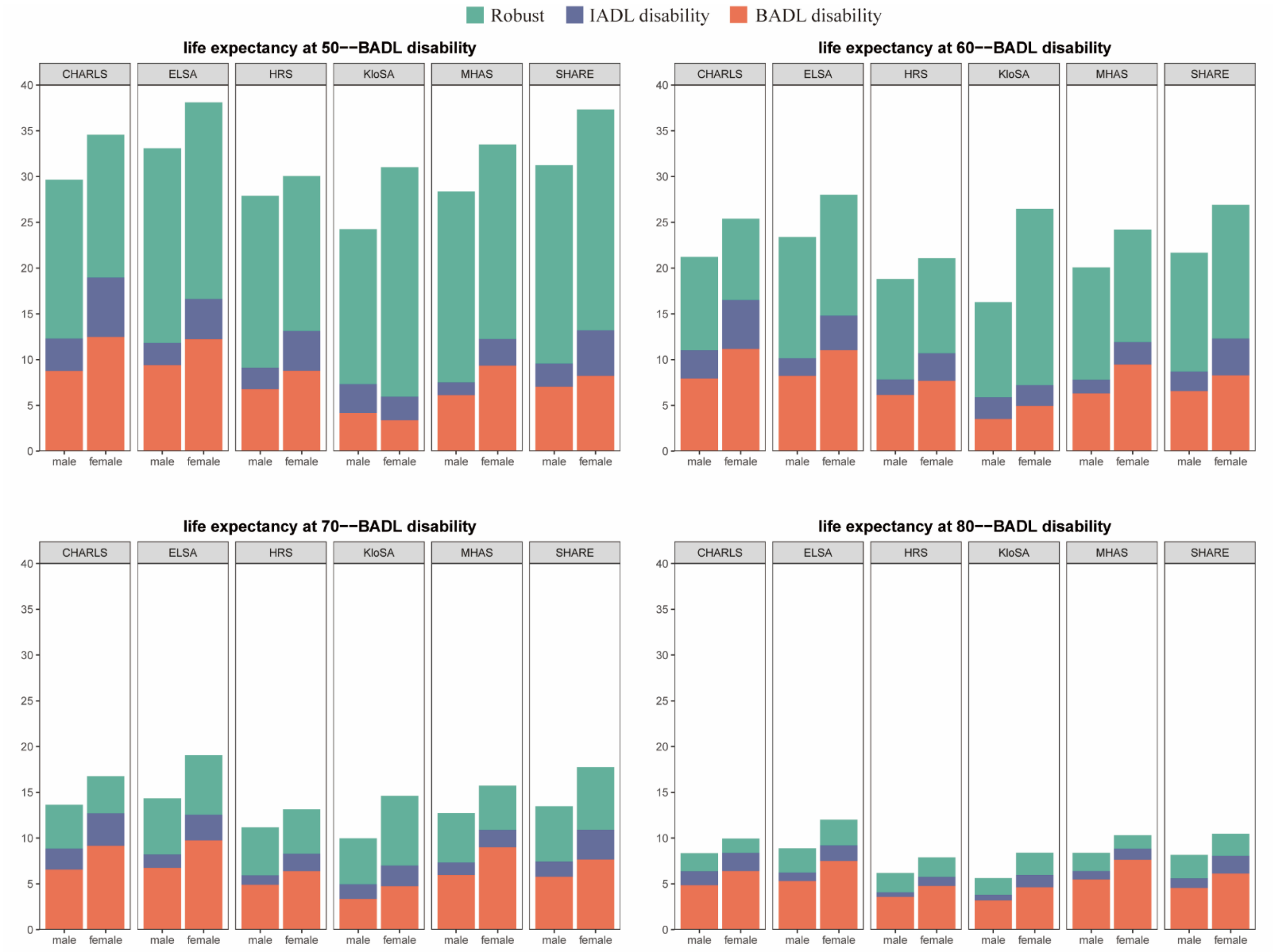

Figure S6. Expected lifespan for individuals with BADL disability at 50, 60, 70, and 80.
